# β-Arrestin 1 (ARRB1) serves as a molecular marker of the peripheral circadian rhythm

**DOI:** 10.1038/s41368-019-0065-y

**Published:** 2019-11-05

**Authors:** Tatsunosuke Tomita, Taisuke Mori, Yoshiaki Onishi

**Affiliations:** 1 Biomedical Research Institute, National Institute of Advanced Industrial Science and Technology (AIST), DBT-AIST International Laboratory for Advanced Biomedicine (DAILAB), Higashi 1-1-1, Tsukuba, Japan; 2 Molecular Pathology Division and Diagnostic Pathology Division, National Cancer Center Research Institute, Tsukiji 5-5-1, Chuo-ku, Tokyo, Japan

**Keywords:** Prognostic markers, Reporter genes, Chromosome conformation capture-based methods

## Abstract

The control of the circadian rhythm is important for health because it regulates physiological functions and is associated with health hazards. We aimed to identify a circadian biomarker of health status in human saliva, since collecting saliva is non-invasive, straightforward, and cost-effective. Among 500 genes potentially controlled by the salivary clock identified using chromatin immunoprecipitation (ChIP) assays, 22 of them showed reasonable transcriptional responses according to a DNA array in a salivary model system. Among these 22 genes, ARRB1, which is expressed in human salivary glands, was also expressed in model HSG cells at the transcriptional and translational levels. The profile of ARRB1 expression in human saliva was circadian, suggesting that ARRB1 could serve as a candidate circadian biomarker in saliva. We compared ARRB1 with other biomarkers in salivary samples from jet-lagged individuals. The circadian profile of ARRB1 reflected the time lag more than the profile of melatonin, whereas the profiles of cortisol and α-amylase did not reflect the time lag. Overall, these findings suggest that salivary ARRB1 could serve as a candidate biomarker that could be used to monitor the internal body clock.

## Introduction

The circadian rhythm is a biological process involving oscillation profiles that are ~ 24 h long and influence various physiological phenomena. The molecular circadian clock controls these phenomena in organisms ranging from cyanobacteria to mammals. The master clock in the suprachiasmatic nucleus (SCN) of the brain hierarchically controls peripheral slave clocks that are found in almost all tissues in mammals.^1,2^ The molecular components common to both the central and peripheral clocks are expressed at all cellular levels. The transcriptional activators, CLOCK and BMAL1, heterodimerize and bind to the E-box responsive elements PER and CRY in the promoter regions of target genes, where they play transcriptional repressive roles in the clock mechanism. Thus, these four components constitute a transcriptional feedback loop. One cycle of this loop takes ~ 24 h and is thus circadian. Therefore, many clock-controlled genes show circadian oscillation in their mRNA expression, resulting in numerous physiological processes that exhibit circadian rhythmicity.^3^ Disorganized rhythmical physiology triggers many pathologies^2^ including sleep–wake cycle disorders,^3^ mood disorders,^4^ and cancer.^5^ In addition, human internal rhythms tend to be more easily disrupted in fast-paced, modern, urbanized global societies. The remedy of such pathologies associated with rhythm disorders or the confirmation of their effects require the internal rhythms of individuals to be monitored. From this perspective, an ideal circadian biomarker is required that can accurately reflect the time of the internal body clock of individuals.

Biological specimens such as blood, urine, and saliva can be sampled for clinical tests. Venous blood is the most frequently sampled specimen because it contains much information about health status and is easily accessible by venipuncture. Therefore, blood is practical for many applications, such as the diagnosis of illnesses, the assessment of physiological status, the monitoring of the health of specific organs, or the screening of genetic conditions. However, phlebotomy is invasive and requires specialized techniques and trained staff. Furthermore, it significantly burdensome for patients when many samples over time are required, such as when monitoring rhythms. In contrast, saliva provides an alternative to blood and other biological specimens because its sampling is non-invasive and less stressful and is thus ideal for sequential sampling. Saliva originates mainly from the major parotid, submandibular and sublingual salivary glands, each of which secretes a characteristic type of saliva with different ionic and protein contents. The main functions of saliva include protecting and maintaining the integrity of the oral mucosa through lubrication, buffering action, and antibacterial and antiviral activities and digestion.^6^ Saliva contains electrolytes, metabolites, hormones, and vitamins, and > 1 000 proteins and peptides, including immunoglobulins and enzymes, according to the findings of proteomic studies. Most of these are found in plasma, but the protein content of saliva is lower than that of blood, meaning that saliva is less complex than blood as a biological matrix and is therefore easier to handle when monitoring compounds of interest. Several physiological and pathological conditions affect the production and content of saliva.^6^ Therefore, saliva is now routinely sampled, for example, to evaluate physiological and biological conditions, such as infection with human immunodeficiency virus (HIV), and for diagnostic purposes and cancer risk assessment. Its flow and composition are regulated by autonomic nervous system activity and controlled in a circadian manner by sympathetic nerve activation by the SCN and biological and peripheral clocks. This means that the timing of saliva collection affects its contents, suggesting that it might be utilized for the measurement of chrono-biomarkers, which are biomarkers that reflect the rhythm of the endogenous biological clock.

Some biomarkers have already been proposed for monitoring circadian rhythms. Melatonin and cortisol are traditional markers of circadian rhythms, and these hormones have been measured as circadian biomarkers. Although useful for monitoring rhythms, these compounds have limitations as biomarkers. Melatonin is a pineal hormone with circadian rhythmic synthesis that peaks at night and is low during the day. This hormone frequently serves in clinical studies as an indicator of internal rhythms. However, the endogenous melatonin content is very low and is found at the pg per mL level in human blood,^7^ and the methods used for precise determinations of hormones with such low values have some limitations. Cortisol has also served as an indicator of internal rhythms in clinical studies.^8,9^ This sterol hormone peaks in the morning and gradually falls throughout the day.^10^ The concentration of plasma or salivary cortisol is higher than that of melatonin, and many detection kits are available for such specimens. However, evaluating fluctuations in cortisol as a specific circadian biomarker is limited because factors such as stress tend to affect cortisol values.^9^

The *BMAL1* promoter contains two recognition motifs for the ROR and REV-ERB orphan nuclear receptors (ROREs). REV-ERBα, which represses *BMAL1* expression, is a major regulator of cyclic *BMAL1* transcription, and RORα (NR1F1) promotes *BMAL1* transcription. Generally, the opposing activities of the orphan nuclear receptors RORα and REV-ERBα are important for the maintenance of circadian clock function. We previously reported that *NR1D1* (*REV-ERBα*) is expressed with a circadian oscillation and was shown to be involved in the circadian transcription of the *BMAL1* gene in a human submandibular gland (HSG) cell line. On the other hand, very little RORα, which is an antagonist of REV-ERBα, is expressed in the HSG, and not much is expressed in NIH 3T3 cells, which serve as a standard model of the circadian system. These results indicate that both BMAL1 and REV-ERBα play important roles in circadian-based transcriptional regulation in the salivary gland. Here, we combined chromatin immunoprecipitation (ChIP) with a DNA microarray (chip) for ChIP-on-chip BMAL1 and REV-ERBα (NR1D1) expression profiling to provide a genome-wide overview of clock-controlled genes in a salivary gland cell model because we hypothesized that the candidate gene is possibly regulated by the salivary gland-specific clock system directly. We then confirmed the candidate gene expression in human salivary glands and analyzed whether the product of the selected candidate gene could serve as a chrono-biomarker in samples of human saliva.

## Results

### Biological clock in HSG cells

**Fig. 1:**
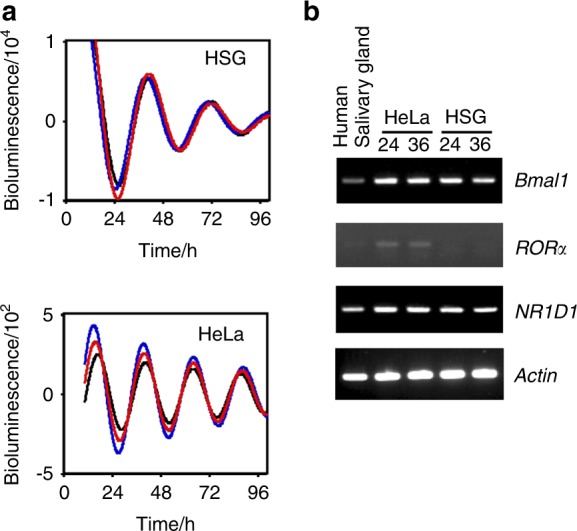
Circadian clock in human salivary gland cells. **a** Circadian transcription of the *BMAL1* gene in HSG and HeLa cells. HSG and HeLa cells were transfected with the *BMAL1* promoter construct (−201 to +24), stimulated with dexamethasone and analyzed using a Kronos AB-2500. The fitted curve data of the detrended results are representative of at least three independent experiments. **b**. Less *RORα* was expressed in HSG cells and human salivary gland tissues. Transcripts from HSG and HeLa cells stimulated with 100 nmol·L^–1^ dexamethasone and human salivary gland tissues were analyzed by RT-PCR

Real-time reporter gene assays showed the obvious circadian oscillation of *BMAL1* promoter activity in HSG and HeLa cells, which showed circadian periods of (31.41 ± 1.03) h and (24 ± 0.89) h, respectively (Fig. 1a). Both *BMAL1*, the oscillation of which is controlled by RORE (ROR α-subunit-binding element), and *NR1D1* were expressed in human salivary gland tissues and in HSG and HeLa cells. However, the amount of expressed *RORα*, which is required for *NR1D1* antagonism, was low in HSG cells and human salivary gland tissues, whereas it was abundant in HeLa cells (Fig. 1b). These results indicate that the systems for circadian gene regulation comprising the core oscillator components *BMAL1* and *NR1D1* were similar in HSG cells and human salivary gland tissues, as we previously reported.^11^

### Identification of clock-controlled genes in salivary cells by ChIP-on-chip analysis

**Fig. 2:**
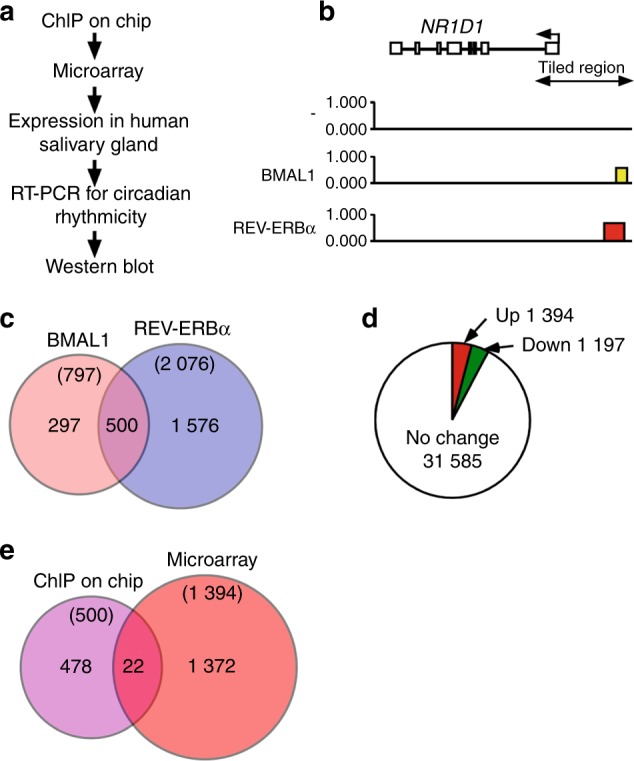
Selection of candidate circadian markers. **a** Flow chart of the selection process. **b** Evaluation of the ChIP-on-chip results for the *NR1D1* region. Transfected HSG cells tagged with BMAL1 and REV-ERBα were harvested at 12 and 24 h after dexamethasone stimulation, and then ChIP was performed using anti-tagged antibodies. Mock-transfected cells without dexamethasone stimulation were processed in the same manner and are indicated as “–”. Precipitated DNA was analyzed using a NimbleGen Human ChIP–chip array, and the *NR1D1* locus and the tiling region are indicated on the map. The red and yellow boxes in the map indicate regions that showed false discovery rates (FDR) of ≤ 0.05 and 0.1 < FDR ≤ 0.2, respectively. **c**. Venn diagram of the gene set intersect between BMAL1-bound cells at 12 h and REV-ERBα-bound cells at 24 h after dexamethasone stimulation in HSG cells. **d** Total RNA was prepared from HSG cells harvested at 12 h and 24 h after dexamethasone simulation, and expression was compared using an Agilent microarray. We found 1 394 and 1 197 genes that were up- and downregulated, respectively, at 12 h compared with their expression 24 h after stimulation. **e** Overlap between genes that might be controlled by the Clock system and genes that were significantly upregulated at 12 h compared to 24 h after stimulation

**Fig. 3:**
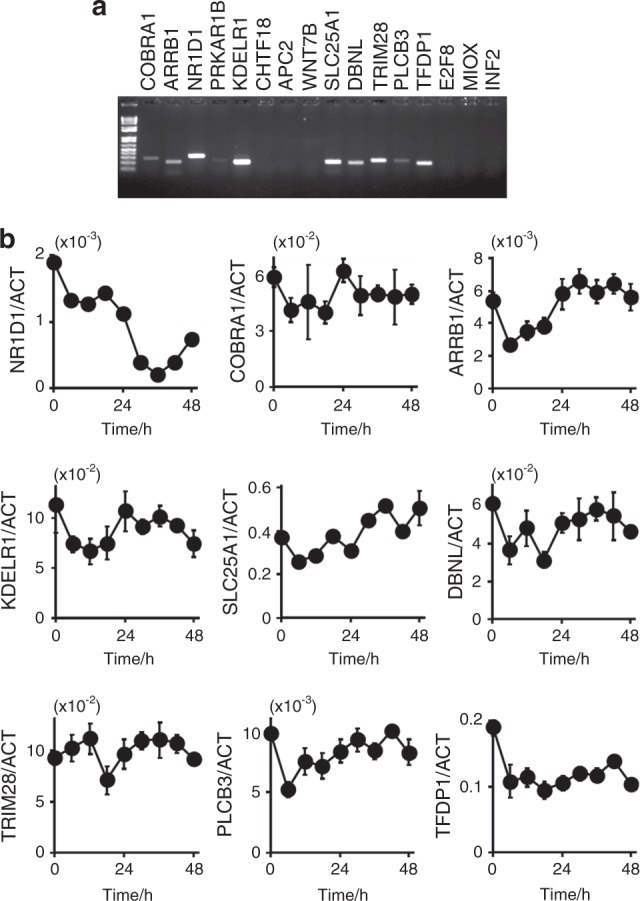
Selection of candidate clones. **a** Expression of clones from the human salivary gland. Transcripts from human salivary glands analyzed by RT-PCR. **b** Rhythmic transcription. Transcripts from HSG cells stimulated with 100 nmol·L^–1^ dexamethasone and analyzed by qRT-PCR

We screened clock-controlled genes to identify a salivary chrono-biomarker (Fig. 2a). Clock-controlled genes in HSG cells were initially selected by ChIP-on-chip assays of the core oscillator components BMAL1 and REV-ERBα. Indeed, *NR1D1* itself is a good candidate chrono-biomarker in HSG cells,^11^ and the ChIP-on-chip assays detected both BMAL1 and REV-ERBα, indicating the integrity of the assay (Fig. 2b). The number of genes that might be individually regulated by BMAL1 and REV-ERBα were 797 and 2 076, respectively, and 500 were regulated by both proteins (Fig. 2c). We then used microarray analysis to determine the differences in gene expression in HSG cells between 12 and 24 h after dexamethasone stimulation. The expression of 1 394 and 1 197 genes at 2 h after stimulation were up- and downregulated, respectively, based on their expression 1 h after stimulation, and the expression of 31 585 genes did not significantly differ between 12 and 2 h after stimulation (Fig. 2d). We combined the ChIP-on-chip and microarray results and selected 22 candidate genes that behaved in a clock-controlled manner, including *NR1D1* (Fig. 2e). We used Reverse transcriptase-polymerase chain reaction (RT-PCR) to analyze the expression of 16 of these genes that were obviously expressed according to the microarray assays in human salivary glands. We detected *COBRA1*, *ARRB1*, *NR1D1*, *KDELR1*, *SLC25A1*, *DBNL*, *TRIM28*, *PLCB3*, and *TFDP1* gene expression in human salivary glands (Fig. 3a). We then examined the circadian rhythmicity of these genes using RT-PCR in HSG cells after dexamethasone stimulation. The circadian rhythms of *NR1D1*, *TRIM28*, *COBRA1*, *SLC25A* and *ARRB1* were obvious, whereas those of *KDELR1*, *PLCB3*, *DBNL* and *TFDP1* were not (Fig. 3b). The phase of the rhythm generated by most candidates reached a trough and a peak at 12 and 2 h, respectively, whereas only that of *TRIM28* showed the opposite pattern.

### Protein detection in salivary cells

**Fig. 4:**
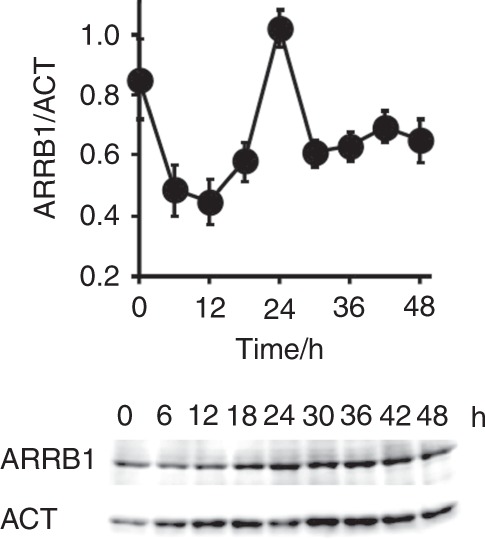
Circadian expression of ARRB1 protein. Protein expression was analyzed by western blotting in HSG cells after stimulation with 100 nmol·L^−1^ dexamethasone. ARRB1, with anti-ARRB1 antibody; ACT, with anti-ACTIN antibody. Values for ARRB1 protein were normalized to those of actin protein, and the peak value was set to 1. Values represent the mean ± SEM of triplicate assays

**Fig. 5:**
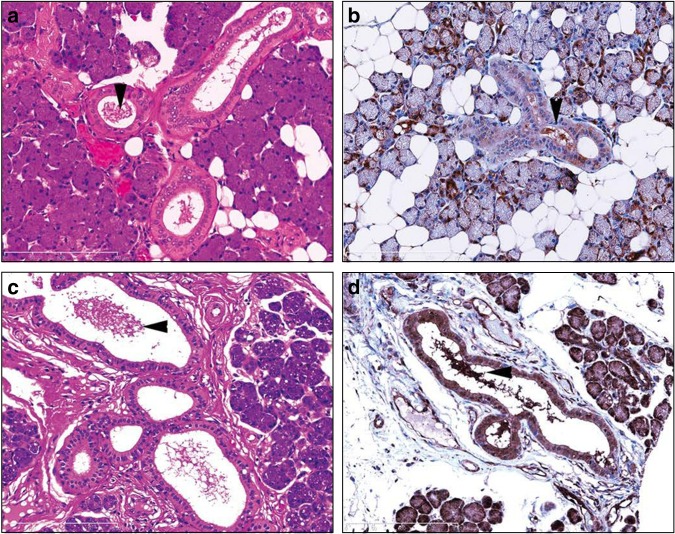
Expression of ARRB1 in normal human salivary glands. ARRB1 was expressed in serous acini, intercalated ducts, striated ducts and excretory ducts in the epithelium of normal parotid glands and submandibular glands. Immunoreactivity to ARRB1 in secreted material. Arrowheads indicate secretions in ducts. Upper panel, acini, intercalated ducts, and striated ducts; lower panel, excretory ducts in the parotid gland. **a**, **c** Haematoxylin and eosin staining; **b**, **d**, ARRB1. Original magnification × 400

We used western blotting to detect the proteins generated by the candidate genes and found that only ARRB1 protein was detectable and expressed in a circadian manner. The rhythmicity of the ARRB1 protein was the same as that determined by RT-PCR, namely, a trough at 1 h and a peak at 2 h (Fig. 4). The expression of ARRB1 protein in the normal human major salivary gland was immunohistochemically detected in > 90% of abluminal (myoepithelial) and luminal (epithelial) cells in acini and between intercalated ducts and excretory ducts (Fig. 5). Less ARRB1 protein was expressed in mucinous than serous acini. Staining identified ARRB1 protein mainly in the membranous or apical portions and rarely in nuclei, and staining varied according to location; staining was more intense in the luminal and abluminal cells of serous acini and intercalated ducts. The material secreted by the ducts was also stained for ARRB1 protein, confirming that this protein is detectable in saliva and thus might serve as a chrono-biomarker.

### Salivary ARRB1 protein

**Fig. 6:**
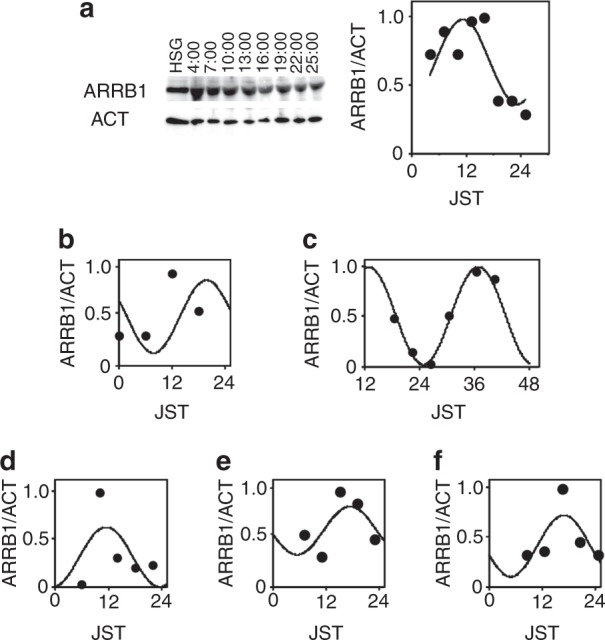
Circadian expression of ARRB1 protein in human saliva. Protein expression in human saliva was analyzed by Western blotting. The amounts of ARRB1 protein were normalized to those of actin protein (ACT), and the peak value was set to 1. Each sample was collected at the indicated time point (JST). The line indicates the fitted cosine curve for the circadian rhythm. **a**–**c**, male adults; **d**–**f**, female adults

**Fig. 7:**
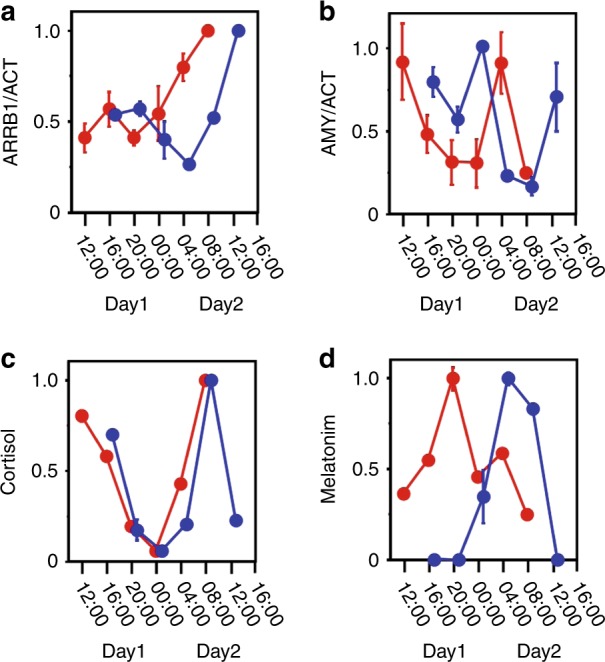
Circadian rhythm in human saliva. Expression of ARRB1 **a** and AMY1A **b** proteins in human saliva analyzed by western blotting. The amounts of ARRB1 and AMY1A proteins were normalized to those of actin protein. The contents of cortisol **c** and melatonin **d** were determined. The peak value was set to 1, and the values are represented as the mean ± SEM of triplicate assays. The red and blue dots represent the samples obtained before and after travel to the region that was 3.5 h behind JST, respectively, (red and blue dots, normal and jet lag samples, respectively). Each sample was collected at the indicated time point (JST), and we denoted “Day 1” as the day on which sampling began both before and after jet lag

Human whole-saliva samples were collected every h and were analyzed by western blotting. Figure 6a shows the clear reaction of ARRB1 protein, which had a ratio to actin that reflected circadian rhythmicity with a peak and a trough at 13:00 and 25:00, respectively, according to the fitted curve. Samples from male and female adults showed individual circadian rhythmicity (Fig. 6b–f). These data support the notion that ARRB1 protein in saliva could serve as a chrono-biomarker. We compared the levels of ARRB1 protein with those of cortisol, melatonin, and amylase, which are biomarkers with established circadian expression.^8,12^ We found that the rhythm of the ARRB1 protein was circadian with a trough at 20:00 (day 1) and a peak at 32:00 (day 2) (Fig. 7a). The respective trough and peak times were 20:00 (day 1) and 28:00 (day 2) for α-amylase (Fig. 7b), the trough for cortisol was reached at 24:00 (day 1) (Fig. 7c) and melatonin peaked at 20:00 (day 1) (Fig. 7d). Compared with samples from subjects with jet lag, the rhythms of ARRB1 protein and melatonin were delayed by 1 h, whereas those of cortisol did not differ (Fig. 7). These results indicate that ARRB1 protein and melatonin reflect individual rhythms and responses to jet lag.

## Discussion

Circadian rhythms are based on cell-autonomous oscillators that maintain gene expression within a period of ~ 24 h. The general molecular mechanism of circadian oscillation in mammals relies on the opposing effects of transcriptional activators and repressors that generate a negative feedback loop.^13^ Among them, CLOCK-BMAL1 is the most important transactivator in the circadian circuit. However, peripheral tissues have their own circadian rhythms, indicating that the mechanism of circadian rhythm regulation is tissue specific. As shown in Fig. 1a, the HSG and HeLa model cells had circadian periods of (31.41 ± 1.03) h and (24 ± 0.89) h, respectively, suggesting the presence of an HSG-specific regulation mechanism. The core oscillator components BMAL1 and REV-ERBα were also rhythmically expressed, indicating that circadian-based transcriptional regulation can be modeled in this peripheral cell type^11^ and that the decreased amount of RORα expression suggests the key role of REV-ERBα in circadian transcriptional regulation in salivary glands. We previously reported that the *NR1D1* gene itself is transcribed in a circadian manner driven by CLOCK-BMAL1 in HSG cells,^11^ suggesting that *NR1D1* is a marker of circadian transcription in salivary glands. Clock genes directly modulate output genes,^14^ and we found 500 genes that might be unique clock-controlled candidate genes in salivary gland model cells. Among these, 22 genes were shown to respond compared with the control cells. We confirmed the expression of these 22 genes in HSG tissues and their circadian rhythmicity in HSG model cells and finally selected the *ARRB1* gene as a candidate circadian biomarker in saliva.

Biomarkers have been included in clinical diagnoses for many years,^15^ and some molecules have been proposed for monitoring the circadian clock. For instance, melatonin is a typical marker of circadian rhythmicity.^7^ The circadian rhythms of blood and salivary melatonin have been investigated in clinical studies^7^ in areas such as gerontology,^16^ psychiatry,^17^ and pediatrics.^18^ The melatonin rhythm is relatively robust to factors such as stress and acute shifts in sleep–wake cycles.^19^ However, the endogenous amount of melatonin in the bloodstream is minuscule (diurnal < 10 pg·mL^−1^), which imposes limitations on its precise determination. Cortisol also shows circadian rhythmicity, with a definite morning surge that triggers adaptive responses to physical activity, as this oscillation is under the control of not only the master clock in the SCN via the hypothalamic–pituitary–drenal axis but also the adrenal internal clock. Endogenous blood and salivary cortisol values range from 50  nmol·L^−1^ to 400 nmol·L^−1^ and (1–25) nmol·L^−1^, respectively.^8^ Although this hormone could be a good biomarker of circadian oscillation, the endogenous content of this hormone is easily affected by acute stress.^9^ In addition to these hormones, protein biomarkers such as salivary α-amylase have been used to determine circadian rhythms. Although the levels of α-amylase clearly oscillate in human saliva, their rhythm did not change under conditions of jet lag because the rhythmic profile reportedly reflects sympathetic nervous system activity and is affected by stressful stimuli.^20,21^ We therefore evaluated the circadian rhythmicity of ARRB1 protein in saliva and its intracellular expression. We identified the ARRB1 gene using ChIP-on-chip and microarray analyses to screen genes controlled by both BMAL1 and REV-ERBα. ARRB1 protein is mainly regulated by clock components in the salivary glands, which are representative peripheral tissues.^11^ Unlike other chrono-biomarkers, ARRB1 protein accurately reflects the endogenous peripheral clock and should therefore be a good biomarker of the peripheral clock, rather than the central clock. Jet lag is considered to be the result of a discrepancy between the central and peripheral clocks;^22^ therefore, markers of both central and peripheral clocks should be useful for clinical diagnoses.

The use of saliva offers some advantages for sequential sampling, such as being non-invasive, and the procedure is straightforward and less stressful for patients. However, saliva can be contaminated with microbes or food particles. Therefore, sampling procedures should be precise and strictly followed to minimize or eliminate contaminating influences. Our volunteers followed the sample collection procedure precisely. Saliva also contains components such as electrolytes and macromolecules that are useful for clinical diagnoses or forensic analyses. For example, DNA extracted from saliva is used for genetic studies to screen for SNPs or mutations. Traditionally, ABO blood typing has been determined from saliva samples. The salivary and blood concentrations of small molecules, such as melatonin and cortisol, are directly related.^23^ For example, salivary and free serum cortisol concentrations are correlated,^24^ and salivary free melatonin concentrations are also thought to be correlated. These small molecules mainly diffuse laterally from blood into saliva through porous capillaries surrounding the salivary glands.^25^ This also allows saliva to be a viable alternative to blood for monitoring the biomarkers discussed above. Saliva contains many proteins with diverse functions that have been identified by proteome analyses. Notably, not only are characteristic proteins, such as α-amylase or immunoglobulin α, actively secreted into saliva, but cytoskeletal proteins, transcriptional and translational factors, proteins involved in cell signaling and energy metabolism, and cell-adhesive factors also are found in saliva. The most critical issue is that the quality of saliva samples is easily affected by many factors. Therefore, appropriate internal controls are needed to normalize saliva quality. We used ACTB protein as an internal control for salivary protein analysis based on published reports.^26,27^ This abundant cytoskeletal protein is used as an indicator of saliva quality for proteomic analyses and is a good biomarker of oral squamous cell carcinoma (OSCC).^28^ The concentrations of ACTB are significantly higher in patients with pre-OSCC than those in healthy individuals. Over 300 000 individuals per year worldwide will develop OSCC, which is a serious local pathology of the oral cavity.^28^ This factor should always be considered because this type of cancer is not clinically rare (2%–3% of all malignancies).

Here, we identified ARRB1 (β-arrestin1) as a candidate circadian biomarker. The negative regulators of G-protein-coupled receptor (GPCR)-mediated signaling were initially identified as β-arrestins that translocate to the cell membrane and bind to agonist-occupied receptors. This process uncouples the receptor from its cognate G proteins and leads to its internalization, which attenuates signalling.^29^ In addition, β-arrestins also function as scaffold proteins and can translocate from the cytoplasm to the nucleus and associate with transcription cofactors.^30^ Our finding that ARRB1 is mainly located in membranous or apical areas and rarely in nuclei is in accordance with these functions. These results suggest that ARRB1 plays important roles in cellular functions, such as cell growth, apoptosis and immune responses, implying that ARRB1 is involved in the circadian regulation of various physiological functions. The light-dependent regulation of the transcriptional activity of retinal arrestin has been reported,^31^ and both the β-arrestin gene and protein are expressed in a circadian manner in ciliary epithelium, where they regulate the circadian rhythm of aqueous flow via the β-adrenergic receptor.^32^ The expression of ARRB1 in saliva peaked in early to mid-afternoon, which was similar to its peak time in ciliary epithelium.^30^ These results support the notion that β-arrestin could function as a circadian salivary biomarker and imply that ARRB1 contributes to the circadian secretion of saliva. Our comparison of salivary samples from individuals whose internal clocks showed a 3.5 h difference from the local time showed that the level of ARRB1 protein reflected a similar time difference as the level of melatonin, whereas the levels of amylase and cortisol did not. This might reflect melanopsin deactivation by ARRB1.^33^

Much knowledge has been obtained about clock genes that control the circadian rhythms involved in physiology and behavior. The effectiveness and toxicity of many drugs vary depending on the dose and when they are administered. Such chronopharmacological phenomena are influenced not only by pharmacodynamics but also by the pharmacokinetics of medications. Thus, knowledge of 24-h rhythms in the context of disease risk together with the evidence of the pharmacokinetic dependence of drugs on 24-h rhythms in terms of effects and safety guide the rationale of pharmacotherapy. Chronotherapy is especially relevant when the risk and/or intensity of disease symptoms vary predictably over time, as is the case for allergic rhinitis, arthritis, asthma, myocardial infarction, congestive heart failure, stroke, and peptic ulcer disease.^34^ The present study identified salivary ARRB1 as a candidate circadian biomarker that might contribute to the future use of chronotherapeutic strategies. ARRB1 may become a more useful biomarker when its effects on physiological function and its underlying mechanisms are elucidated. However, further investigation is required to confirm its effects.

## Materials and methods

### Cell culture

Both HSG and HeLa cells were cultured in Dulbecco's modified Eagle medium (DMEM) supplemented with 10% fetal bovine serum (FBS) and a mixture of penicillin and streptomycin in a humidified incubator at 37 °C in a 5% CO_2_ atmosphere.

### Tissue samples

Formalin-fixed, paraffin-embedded specimens were collected from 14 normal major salivary glands (parotid, *n* = 8; submandibular glands, *n* = 6) for pathological diagnosis at the National Cancer Center Hospital in Tokyo. Written informed consent was obtained from the patients, and the Ethics Committee of the National Cancer Center Hospital, Tokyo, Japan, reviewed and approved the study protocol (Approval #2010-075).

### Real-time reporter gene assays

The real-time reporter gene assays were performed as described.^11^ A reporter plasmid based on pGL3-dLuc^35^ was transfected using HilyMax (Dojindo Molecular Technologies Inc., Kumamoto, Japan). After a 24-h incubation, the cells were stimulated with dexamethasone (100 nmol·L^–1^) for 2 h and then incubated with DMEM containing 0.1 mmol·L^–1^ luciferin (Promega Corporation, Fitchburg, WI, USA), 25 mmol·L^−1^ HEPES (pH 7.2) and 10% FBS. The bioluminescence was measured and integrated for one min at 10-min intervals using a Kronos AB-2500 (ATTO Technology Inc., Amherst, NY, USA). The data were detrended by the subtraction of the best fit line and subsequent fitting to a sine wave to determine the length of the circadian period, as previously described.^36^

### RT-PCR

Real-time PCR was performed as described.^35^ Total RNA was prepared using acid guanidinium thiocyanate–phenol–chloroform and human salivary gland total RNA (Clontech). The first strand cDNA was synthesized from RNA (1 μg) using PrimeScript RT-PCR kits (Takara Bio Inc., Kusatsu, Shiga, Japan). Then, the PCR fragments were resolved by electrophoresis with 2% agarose gels and visualized by ethidium bromide staining. Real-time quantitative RT-PCR was performed using a LightCycler (Roche Diagnostics GmbH, Mannheim, Germany) with a LightCycler-FastStart DNA Master SYBR Green I kit (Roche Diagnostics). The primer sequences used were as follows: *BMAL1*, 5'-AAGACTTCCCCTCTACCTGCTC -3' and 5'-AACTACATGAGAATGCAGTCGTC-3'; *ACTIN*, 5′-TACGCCAACACAGTGCTGTCTG-3′ and 5′-TTTTCTGCGCAAGTTAGGTTTTGTC-3′; *RORα*, 5′-GATGTATTTTGTGATCGCAGCGATG-3′ and 5′-TACGGCAAGGCATTTCTGTAATCG-3′; *Rev-erbα/NR1D1*, 5′-CCTGGACTCCAACAACAACAC-3′ and 5′-ACACTCGGTTGCTGTCCTCCA-3′; *COBRA1*, 5′-AAGTCGCCAAAGGTCTCCAC-3′ and 5′-TCTTCAAGGAGCCCAAGATG-3′; *ARRB1*, 5′-AGAAGCCTCTCTGGATAAGG-3′ and 5′-GTAGACCTTGCAGAACGTCG-3′; *PRKARB1*, 5′-CGTGTCCTACGTCAGGAAG-3′ and 5′-ACTCTCCGTTCACGTACAC-3′; *KDELR1*, 5′-CACTCTACAACACGTGTATG-3′ and 5′-TAGATGGAGAAGGTCCAGAG-3′; *CHTF18*, 5′-ACACGTGATTGCGCGTCAC-3′ and 5′-ATGCTCAGGAGGACGTTGATG-3′; *DBNL*, 5′-ATGTCCACCACCTCCATCTC-3′ and 5′-TTCTGCCTGCACCAGACATG-3′; *TRIM28*, 5′-ATGCGTGATAGTGGCAGCAAG-3′ and 5′-GCAATAGACAGTACGTTCAC-3′; *PLCB3*, TGCAGGATGACACAGCCAAG-3′ and 5′-AATTTGAGGCCACAGGATTCC-3′; *TFDP1*, 5′-GCTCAGGAATGTCAGAACTTAG-3′ and 5′-GCAGGTGGATGACTGAGTTG-3′; *E2F8*, 5′-TGAGGCCAAAGACTGTATAC-3′ and 5′-CACGATATCGTAAATGCGTCG-3′; *MIOX*, 5′-ACCTCCAGGATCCTCGATAC-3′ and 5′-AACCGGATCATGTAGAAAGC-3′; *INF2*, 5′-AGCTGCGGAACGAGTTTATC-3′ and 5′-AGCAGCTCACCTTGTGGAAC-3′. We generated an authentic template comprising PCR products cloned into the pGEM-T Easy vector (Promega). The relative expression was evaluated using LightCycler software, version 3.5.

### ChIP-on-chip analysis

Chromatin immunoprecipitation assays were performed as described.^35^ In brief, HSG cells were transfected with Myc-tagged REV-ERBα or Flag-tagged BMAL1, incubated for 24 h and then stimulated with 100 nmol·L^−1^ dexamethasone for 2 h. The protein-DNA complexes were cross-linked with 1% formaldehyde for 10 min at room temperature. The cells were lysed, then the chromatin was fragmented and immunoprecipitated using anti-Myc antibody (Roche Diagnostics), anti-Flag antibody (Sigma, St. Louis MO, USA) and protein A/G Sepharose (Santa Cruz Biotechnology Inc., Santa Cruz, CA, USA). The immunocomplexes were eluted with 1% SDS and 0.1 mol·L^−1^ NaHCO_3_, then the cross-linking was reversed by heating the immunocomplexes at 65 °C for 4 h. The cross-linking of the DNA input samples was similarly reversed. We purified and amplified the DNA from the samples using WGA amplification kits (Sigma) to obtain sufficient DNA for hybridization. The amplified DNA was sent to NimbleGen Systems Inc. (Madison, WI, USA) for labeling and array hybridization on a NimbleGen Human ChIP–chip 3 x 720K RefSeq Promoter Array. The results were analyzed, and the peaks were identified at Roche NimbleGen Inc. (Madison, WI, USA). The binding peaks were detected by searching for at least four probes with signals above the specified cutoff values, ranging from 15% to 90% with a 500-bp sliding window. The cutoff values were ratios (%) of the hypothetical maximum and the mean ± 6 SD. The ratios were randomized 20 times to evaluate the probability of false positives. Each peak was then assigned a false discovery rate (FDR) based on the number of peaks that continued to exceed the cutoff values after 20 randomizations. Binding peaks with an FDR ≤ 0.2 were considered representative of strong/true binding regions.

### Microarrays

Total RNA was prepared from HSG cells and stimulated with dexamethasone using acid guanidinium thiocyanate–phenol–chloroform. Cy3-labeled cRNA was synthesized from 250 ng of total RNA using an Agilent Low RNA Input Linear Amplification Kit PLUS, One-Color (Agilent Technologies, Santa Clara, CA, USA) and hybridized at 65 °C for 17 h to an Agilent Whole Human Genome Microarray 4x44K. The microarrays were then washed and scanned using an Agilent DNA microarray scanner. The intensity of each scanned feature was quantified using Agilent Feature Extraction Software with background subtraction. The data were normalized using Agilent GeneSpring GX ver. 11.0.2. (per chip normalization, 75% shift; per gene normalization, none). All analyses were performed at DNA Chip Research Inc. (Tokyo Japan).

### Western blotting

Both sodium dodecyl sulfate polyacrylamide gel electrophoresis (SDS–PAGE) and western blotting were performed as previously described.^35^ The collected saliva was centrifuged at 10 000 r·min^−1^ for 10 min to remove the insoluble material. The supernatant proteins were resolved by 9% SDS–PAGE and transferred to polyvinylidene difluoride membranes (GE Healthcare, Amersham, UK). Non-specific binding was blocked with EzBlock Chemi buffer (ATTO). The proteins were probed with an anti-ARRB1 antibody (Abcam plc., Cambridge, UK), an anti-actin antibody (Santa Cruz Biotechnology) or an anti-amylase antibody (Santa Cruz Biotechnology) and then incubated with horseradish peroxidase-conjugated anti-rabbit or mouse IgG (Upstate Biotechnology Inc., Charlottesville, VA, USA). The immunoreactive proteins were visualized using ECL (GE Healthcare) according to the manufacturer’s instructions.

### Immunohistochemical analysis

Tissue microarray blocks were cut into 4-μm-thick sections, deparaffinized and stained with haematoxylin–eosin, and the sections were then immunohistochemically stained with a primary arrestin1 antibody that was diluted 1:200 (Abcam; E274). The sections were immersed in 0.3% hydrogen peroxide for 15 min to block endogenous peroxidase activity. All staining was automated (Dako GmbH., Jena, Germany) according to the vendor’s protocol, and the proteins of interest were detected using ChemMate EnVision reagent (Dako).

### Preparation of human saliva samples

Saliva from male and female adults (30–55 years old) was collected by unstimulated passive drool at the indicated times, none of which were within 60 min or 12 h of consuming a major meal or alcohol, respectively. The mouth was thoroughly rinsed with water to minimize the effects of lowering the sample pH and influencing bacterial growth. Samples were collected 10 min later and were immediately stored at −20 °C. The jet lag samples from 50-year-old males were collected after they returned from a one-week stay in a region that is 3.5 h behind Japanese Standard Time (JST). The mucins and precipitants were removed by centrifugation of the thawed saliva samples before analysis.

### Cortisol and melatonin content of human saliva

Cortisol and melatonin were measured using Cortisol ELISA (Cayman Chemical Company, Ann Arbor, MI, USA) and Salivary Melatonin ELISA (Salimetrics LLC, Carlsbad, CA, USA) kits according to the manufacturers’ protocols.
